# Cathepsin K induces platelet dysfunction and affects cell signaling in breast cancer - molecularly distinct behavior of cathepsin K in breast cancer

**DOI:** 10.1186/s12885-016-2203-7

**Published:** 2016-03-01

**Authors:** Sheila Siqueira Andrade, Iuri Estrada Gouvea, Mariana Cristina C. Silva, Eloísa Dognani Castro, Cláudia A. A. de Paula, Debora Okamoto, Lilian Oliveira, Giovani Bravin Peres, Tatiana Ottaiano, Gil Facina, Afonso Celso Pinto Nazário, Antonio Hugo J. F. M. Campos, Edgar Julian Paredes-Gamero, Maria Juliano, Ismael D. C. G. da Silva, Maria Luiza V. Oliva, Manoel J. B. C. Girão

**Affiliations:** Departments of Gynecology of Universidade Federal de São Paulo, São Paulo, SP 04024-002 Brazil; Biophysics of Universidade Federal de São Paulo, São Paulo, SP 04024-002 Brazil; Biochemistry of Universidade Federal de São Paulo, São Paulo, SP 04024-002 Brazil; Charitable Association of Blood Collection – COLSAN, São Paulo, SP 04080-006 Brazil; Department of Pathology, AC Camargo Hospital Biobank, A C Camargo Cancer Center - Antonio Prudente Foundation, São Paulo, SP 01509-010 Brazil; Department of Gynecology, Cellular Gynecology Laboratory, Universidade Federal de São Paulo, Rua Napoleão de Barros, 608, CEP 04024-002 São Paulo, Brazil

**Keywords:** Cathepsin K, Platelets, Breast cancer, Protease activated receptors

## Abstract

**Background:**

Breast cancer comprises clinically and molecularly distinct tumor subgroups that differ in cell histology and biology and show divergent clinical phenotypes that impede phase III trials, such as those utilizing cathepsin K inhibitors. Here we correlate the epithelial-mesenchymal-like transition breast cancer cells and cathepsin K secretion with activation and aggregation of platelets. Cathepsin K is up-regulated in cancer cells that proteolyze extracellular matrix and contributes to invasiveness. Although proteolytically activated receptors (PARs) are activated by proteases, the direct interaction of cysteine cathepsins with PARs is poorly understood. In human platelets, PAR-1 and −4 are highly expressed, but PAR-3 shows low expression and unclear functions.

**Methods:**

Platelet aggregation was monitored by measuring changes in turbidity. Platelets were immunoblotted with anti-phospho and total p38, Src-Tyr-416, FAK-Tyr-397, and TGFβ monoclonal antibody. Activation was measured in a flow cytometer and calcium mobilization in a confocal microscope. Mammary epithelial cells were prepared from the primary breast cancer samples of 15 women with Luminal-B subtype to produce primary cells.

**Results:**

We demonstrate that platelets are aggregated by cathepsin K in a dose-dependent manner, but not by other cysteine cathepsins. PARs-3 and −4 were confirmed as the cathepsin K target by immunodetection and specific antagonists using a fibroblast cell line derived from PARs deficient mice. Moreover, through co-culture experiments, we show that platelets activated by cathepsin K mediated the up-regulation of SHH, PTHrP, OPN, and TGFβ in epithelial-mesenchymal-like cells from patients with Luminal B breast cancer.

**Conclusions:**

Cathepsin K induces platelet dysfunction and affects signaling in breast cancer cells.

**Electronic supplementary material:**

The online version of this article (doi:10.1186/s12885-016-2203-7) contains supplementary material, which is available to authorized users.

## Background

Proteases from epithelial, myoepithelial, stromal, and tumor cells become activated during neoplastic progression and can display causal roles in tumor growth, migration, invasion, angiogenesis, and metastasis [1–5]. However, identification of the exact tissue of origin, temporal release, and activation is not fully established.

Human cysteine cathepsins (Cat) are proteases that are highly up-regulated in a wide variety of cancers. Active forms of cathepsins are localized in endosomal or lysosomal vesicles, cell membranes, and/or secreted and localized in pericellular environments as soluble enzymes that are involved in cleaving the extracellular matrix proteins, laminin and type IV collagen, and cell-adhesion proteins such as E-cadherin and matricellular proteins [2, 6–8].

Proteolytically activated receptors (PARs) constitute a family of G-protein-coupled receptors that are activated during one of several protease-generating pathways in humans, such as inflammatory, fibrinolytic, and hemostatic pathways and cancer; PARs are also activated by proteases, particularly thrombin, via a specific proteolytic cleavage of their amino-terminal exodomain [9–12]. The PAR-mediated mitogenic pathway regulates tumor cell growth and can promote tumor cell invasion [13]. Several examples of PARs up-regulation and their potential in activating proteinases in tumor tissues, including breast, prostate, and colon cancer, and malignant melanomas, have been reported [11, 14]. In addition, abnormalities in blood coagulation are common in malignant tumors [15]. Tumor cells have platelet aggregating activity that occurs through different mechanisms including the activation of PARs. PAR-1 and −4 show the highest expression in human platelets among the four currently identified PARs [16, 17]. PAR-3 shows the lowest expression and appears to be preferentially expressed in cells of hematopoietic origin, suggesting a function distinct from that of PAR-1, which is the major receptor involved in thrombin-mediated platelet activation [18]. Furthermore, PAR-3 has been shown to be a major thrombin receptor in mouse platelets; however, its role in humans remains uncharacterized [11, 19–21]. In this scenario, the link between human cysteine cathepsins and platelet functions in malignant conditions is underexplored.

The cysteine cathepsins used in our study, K, L, V, S, and B, are particularly attractive drug targets [8, 22]. Cat K is of relevant interest because it is a cysteine protease implicated in bone remodeling, breast cancer progression, and other diseases [23–26].

We investigated platelet aggregation using washed platelets, which enabled the identification of PARs involved in this process, to determine the role of cathepsins in human platelet aggregation and the detailed triggering signal produced by cathepsins on platelets. In addition, we examined whether Cat K alone, which was activated in epithelial-mesenchymal cells from women with breast cancer or its co-culture with Cat K activated human platelets, could directly affect the expression of ligands in the Hedgehog signaling pathway. The expression of these ligands, reported as an aberrantly activated and proto-oncogenic pathway in breast cancer, is related to bone metastasis markers.

## Methods

A detailed material and methods section can be found in the Additional file 1.

### Collection of human platelets

Human platelets were obtained from donors within the Charitable Association of Blood Collection – COLSAN in São Paulo, SP, Brazil. The study was carried out in accordance with the Declaration of Helsinki and approved by the Institutional Ethics Review Board (Ethics Committee in research of the Federal University of São Paulo - CEP1917/11) from the São Paulo Federal University/São Paulo Hospital (UNIFESP-HSP). A written informed consent was signed by each patient, who volunteered to participate before the study start.

### Enzyme preparation

The K, L, S, V, and B cysteine proteinase cathepsins were purified as previously described [27]. Papain was obtained from Sigma (St Louis, MO, USA) and Human α-thrombin (200 NIH units/mg) was purchased from Helena Laboratories (Beaumont, Texas, USA).

### Platelet aggregation

Pooled venous blood was collected from healthy donors to obtain platelet-rich plasma (PRP); washed platelets were prepared as previoulsy described [28]. Agonist solutions containing each of the cysteine proteinases cathepsins were added (separately one by one) to the washed platelets aliquot: Cat K (20 nM), cathepsins L, V, S, and B (all enzymes at 0.2 μM), and papain (1.6 μM). α-Thrombin (1.0 UNIH/500 μl), 0.2 μM activating peptides-PAR1 (AP-PAR-1), or 200 μM AP-PAR-4 were used as agonists for aggregation in washed platelet suspensions. Enzymes, preincubated with cysteine inhibitors (E-64 (5 μM), LWMK (1.0 μM), and HWMK (1.0 μM)), and platelets pretreated with PAR-3 antibody and PAR-1 (SCH 79797), and PAR-4 (trans-cinnamoyl-YPGKF-NH_2_) antagonists were tested as blockers for Cat K-induced platelet aggregation.

### Peptide synthesis

FRET peptide substrates containing sequences from PAR-1, −3, and −4 proteinase-activated receptors were synthesized as previously described [29]. The molecular mass and purity of synthesized peptides were assessed by analytical HPLC and MALDI-TOF using a Microflex -LT mass spectrometer (Bruker – Daltonics, Billerica, MA, USA). Stock solutions of peptides were prepared in dimethyl formamide, and their concentrations were determined spectrophotometrically using the 2,4-dinitrophenyl group (Dnp) molar extinction coefficient of 17.300 M^−1^cm^−1^ at 365 nm.

### Hydrolysis of PARs derived FRET substrates by human cathepsins cysteine proteinases

The hydrolysis of FRET peptides by human cysteine cathepsins was performed as previously described [30]. The V_max_ and K_M_ kinetic parameters were determined from the initial rate measurements at 8–10 substrate concentrations between 0.15 and 5 K_M_. Enzyme concentrations were chosen so that less than 5 % of the substrate was hydrolyzed during the assay. The reaction rate was converted to micromoles of hydrolyzed substrate per minute based on a calibration curve obtained by the complete hydrolysis of each peptide. The data were fitted with the respective standard errors to the Michaelis-Menten equation using the GraFit software (Erithacus Software Limited). At least duplicate data were collected in all assays; the error values were less than 10 % for each of the obtained kinetic parameters.

### Extraction and real-time reverse transcription-PCR analysis

Total RNA was isolated from Cat K treated and untreated human platelets to detect PAR-3 expression. cDNA was generated using the ImProm-II^TM^ reverse transcription kit (Promega, Madison, WI, USA). The quantitative RT-PCR performance data is presented in the Additional file 1.

### Protein preparation and immunoblot analysis

Washed platelets were treated with cathepsins cysteine proteinases K (20 nM), L, V, S, and B (all enzymes at 0.2 μM), papain (1.6 μM), and α-thrombin (0.001 UNHI/mL) for 10 min at 37 °C for the detection of PARs −1, −3, and −4 and extraction of signaling phospho proteins into platelets (p-PKC, p-SOD, p-Src family, p-FAK, and p-p38). Whole cell lysates were collected in 20 mM Tris buffer containing 300 mM NaCl, 2 mM EGTA, and 2 % Nonidet P-40. Co-culture was performed for the detection of Src, p-Src, FAK, p-FAK, PTHrP, Cat K, OPN, SHH, and TGFβ. Total protein (60 μg) was assessed by SDS-PAGE gel and transfer to nitrocellulose membranes. See Additional file 1 for details.

### Platelet activation

The activation of washed human platelets was assessed using fluorescence-activated cell sorting (FACS) by Accuri C6 (BD-Biosciences, San Jose, CA, USA). Human platelets were detected using CD61-FITC labeling (GPIIIa – integrin β3) as a specific marker. The light scatter and fluorescence channels were set at a logarithmic gain. PRP cells (5 × 10^7^ cells/mL) were treated with α-thrombin (0.001 U/mL), cathepsins K (20 nM), L, V, S, and B (all enzymes at 0.2 μM), and papain (1.6 μM) for 10 min at 37 °C. The exposure of phosphatidylserine was monitored using annexin-V-PE labeling according to the manufacturer’s instructions (BD Pharmingen, San Diego, CA, USA) and identified by cytofluorometry. Platelet lysis, determined by lactate dehydrogenase release, was not observed. We used apyrase (5.0 UNIH/mL), an ATP-diphosphohydrolase, to prevent ADP amplification in platelet activation.

### Calcium mobilization by confocal microscopy

Washed human platelets were incubated with 4 μM Fluo-4/AM (Molecular probes, Invitrogen, USA) at room temperature for 30 min. After incubation, washed platelets were centrifuged at 141 × g for 5 min for subsidence to coverslips (12 mm diameter). Briefly, platelets were stimulated with cathepsins K (20 nM), L, V, S, and B (all enzymes at 0.2 μM), papain (1.6 μM), and α-thrombin (1.0 U/mL); Fluo-4/AM was excited with an argon laser (λ_Ex_ = 488 nm) and light emission was detected using a Zeiss META detector (λ_Em_ = 500–550 nm). See Additional file 1 for details.

### Tissue isolation and cell culture

Thrombin receptor (ThrR−/−) knockout mice were obtained from CEDEME at the Federal University of São Paulo (UNIFESP, São Paulo, Brazil). All mice were housed and handled in accordance to the guidelines proposed by the Brazilian Council for Animal Experimentation (COBEA) and approved by the Research Ethics Committee of the Federal University of São Paulo, São Paulo, Brazil, under number 0858/10. The isolation and preparation of fibroblast cell lines were adapted from Trejo et al. [31].

Mammary tissues were obtained from informed volunteers in the mastology Unit/Department of Gyne- cology at the Federal University of São Paulo/São Paulo Hospital (UNIFESP/HSP) with approved Ethics Committee in research of the Federal University of São Paulo (CEP1917/11). A written informed consent was signed by each patient, who volunteered to participate before the study start. Primary mammary cell culture was prepared from biopsies from 15 women who had luminal B subtype breast cancer (each tumor was analyzed for ER/PR/HER2 and Ki-67 high (≥14 % cells positive) status) and an average platelet count of 635 (× 10^3^/μL). Tissue samples were rinsed, minced into small pieces, and digested with collagenase IA (0.05 mg/ml) for 16 h at 37 °C. After this incubation, samples were centrifuged (differential centrifugation) to separate stromal and epithelial cells. Cells were placed in primary flasks (Becton–Dickinson Labware, Le Pont de Claix, France) and cultured in Dulbecco’s modified Eagle’s medium (DMEM)/F-12 without phenol red and with 10 % fetal bovine serum (FBS) supplemented with 10 μg/mL insulin, 20 ng/mL epidermal growth factor, 0.5 μg/mL hydrocortisone, 100 ng/mL cholera toxin, and 1 % penicillin/streptomycin in a humidified incubator at 37 °C and 5 % CO_2_. After 24 h incubation, non-adherent cells were washed out, and adherent epithelial cells were characterized by immune staining with FITC-labeled anti-cytokeratin, E-cadherin, N-cadherin, PAI-1, claudin, and Cy3-labeled anti-vimentin antibodies.

### Zymography

Conditioned media from primary cells (epithelial and epithelial-mesenchymal-like transition phenotype) were collected and centrifuged to remove cellular debris. Volumes of conditioned media normalized to the number of cells were subsequently mixed with the sample buffer and loaded onto a 7.5 % acrylamide/bisacrylamide separating gel containing 0.02 % (*w/v*) gelatin. After electrophoresis, the gel was incubated in 2.5 % Triton X-100, rinsed in distilled water, and incubated for 16 h at 37 °C in buffer containing 50 mM Tris pH 7.6, 20 mM NaCl, 5 mM CaCl_2_, and 2 μM ZnCl_2_. The gel was stained with 0.1 % Coomassie blue R-250, 30 % methanol, and 10 % acetic acid, and destained in the same solution without Coomassie blue.

### Treatment of tumor cells with Cat K activated platelets

Cells were seeded in DMEM-F12 (as see above) and incubated overnight. After this incubation, a total of 200,000 platelets/μl in fresh medium was immediately treated with Cat K (20 nM). Platelets were removed for analysis, and epithelial-mesenchymal-like cells and epithelial cells were washed twice with PBS. Western blot was performed to identify the proteins involved in the amplification of the Hedgehog pathway (Src, SHH, PTHrP, Cat K, OPN, and TGFβ).

### Phenotypic analysis of breast cancer cells and Cat K activated platelets

Epithelial-mesenchymal-like transition cells co-cultured with washed human platelets activated by Cat K were submitted to phenotypic analysis. Platelets were removed for analysis, and epithelial-mesenchymal-like cells were washed twice with PBS and adjusted to 1 × 10^6^/mL. Cell suspensions were incubated for 30 min at room temperature and in the dark with CD44-phycoerythrin (PE) and washed platelets were incubated with P-selectin-fluorescein isothiocyanate (FITC) for labeling according to the manufacturer’s instructions (BD Pharmingen, San Diego, CA, USA). Cells were fixed with 4 % paraformaldehyde and analyzed using a Becton Dickinson and Company flow cytometer (Accuri C6™).

### Statistical analysis

The results from the in vitro studies are presented as means of three independent experiments. The statistical analysis was performed using GraphPad PRISM5.0 (La Jolla, CA). Briefly, the Student’s t-test was used to compare means between two independent groups, whereas one-way ANOVA followed by the Tukey’s post-test, was used to compare means between two or more independent groups. Two-way ANOVA was used to compare group means influenced by two independent factors. The error bars represent the SEM in some of the figures, and SD in others. The level of p ≤ 0.05 was accepted as significant.

## Results

### Cat K induced aggregation in human platelets

**Fig. 1:**
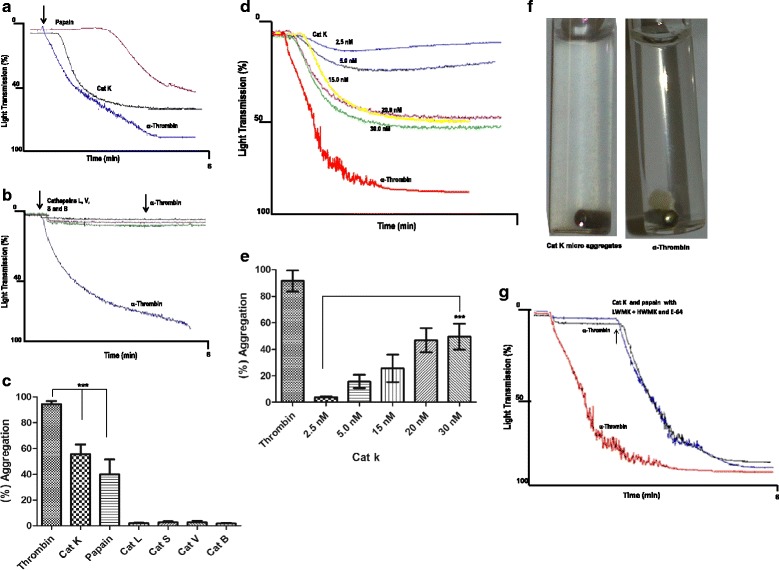
Cat K-induced human platelet aggregation. Dose-response curve and effect of LWMK, HWMK, and E-64 under cat K activity. The washed human platelets (3.0 x 105/mL) function was measured at baseline and stimulus was added after 30 s. (**a**) Effect of cat K (20 nM) and papain (1.6 µM) on platelet aggregation. (**b**) Negative effect of cathepsins L, V, S, and B (at a median effective dose - ED50 - of 0.2 µM) on platelets showing typical tracing results, α-thrombin (1.0 UNIH/500 µL) was used as the agonist control. (**c**) The bar graph shows percentage of aggregation. Data are presented as means ± SEM (***p<0.0001). Traces show one typical experiment out of at least six. (**d**) Solutions containing different concentrations of cat K (2.5 to 30 nM) were added to platelets and aggregation was measured as described. Representative traces showing the typical aggregation induced by cat K at different concentrations and α-thrombin (1.0 UNIH/500 µL). (**e**) Concentration-response bar graph expressed as percentage of aggregation (***p<0.001). (**f**) Clot aggregate analyses of human platelets treated with cat K (20 nM) and α-thrombin (1.0 UNIH/500 µL). (**g**) Inhibition of human cat Kactivity (black line) and papain (blue line) on human platelet aggregation by E-64 (5 µM), LWMK (1.0 µM) + HWMK (1.0 µM)

The human cysteine cathepsins’ ability to induce platelet aggregation was evaluated through the individual addition of K, L, V, S, and B cathepsins, at final concentrations of up to 20 nM, to washed human platelets suspensions; aggregation curves were recorded for 6 min in an aggregometer. Papain (1.6 μM) and α-thrombin (1.0 UNHI/mL) were used as agonist controls. The L, V, S, and B cathepsins at a median effective dose (ED_50_) of 0.2 μM failed to induce any measurable aggregation while Cat K showed an effect of up to 40 % aggregation, similar to that observed with papain (Fig. 1a-c).

Similar aggregation patterns were observed in all assayed concentrations; however, the aggregation magnitude showed a linear correlation with Cat K concentrations in the 2.5–20 nM range; further increase in concentrations, up to 40 nM, did not show improved aggregation levels (Fig. 1d, e). The maximal extent of aggregation obtained at 20 nM was 46.8 ± 10 %.

Moreover, the type of aggregation clot induced by Cat K differed from the characteristic clot induced by α-thrombin. The Cat K lag phase was followed by a second aggregation wave that induced the formation of micro-aggregates, whereas α-thrombin showed a biphasic curve with the formation of a tight and irreversible characteristic platelet clot (Fig. 1f).

The proteolytic nature of this activation was confirmed by the complete inhibition of platelet aggregation when Cat K (20 nM) and papain (1.6 μM) were preincubated with the irreversible cysteine proteinase inhibitor E-64 (5 μM), or the human high and low molecular weight kininogen (HMWK and LMWK at 1.0 μM; Fig. 1g). The level of platelet aggregation observed when α-thrombin was further added to the system confirmed that these three cysteine protease inhibitors did not interfere with the platelets’ ability to respond to α-thrombin activation (Fig. 1g).

Table 1 shows the in vitro kinetic parameters of the hydrolysis of synthetic FRET substrates, and their cleavage sites derived from sequences that span the cleavage sites for PARs −1, −3, and −4 activations by Cat K, papain, and α-thrombin. The peptide derived from the PAR-2 sequence was not included in this study because PAR-2 is not present in platelets (Additional file 1: Table S1). Z-F-R-MCA, the standard substrate for papain and Cat K, was assayed as the positive control for both enzymes [7, 27, 32–34]. All three PAR substrates were cleaved by both cysteine proteinases with k_cat_/K_M_ values in the same order of magnitude as Z-F-R-MCA. The PAR-1 substrate was only hydrolyzed between Leu and Qln-EDDnp, a peptide bond not available in the PAR-1 sequence. Cat K and papain effectively cleaved PAR-3 and PAR-4 peptides at the Lys-Thr and Arg-Gly bonds, mimicking the α-thrombin activity on this substrate and supporting both enzymes as PAR-3 and PAR-4 agonists. The PAR-3 peptide was hydrolyzed by Cat K only at the Lys-Thr peptide bond forming the tethered ligand sequence of PAR-3 TFRGAP; nevertheless, the presence of other papain cleavage sites on PAR-3 indicates a possible antagonistic effect. The kinetic hydrolysis parameters and PARs-1, −3, and −4 peptides cleavage sites used by cat L, S, V, and B are shown in Table 2.

**Table 1:** Kinetic parameters for the hydrolysis of the FRET peptides derived from sequences that span the cleavage sites for activation of protease-activated receptors (PAR) 1, 3 and 4 by cat K and papain

Enzyme		Peptide	Abz-peptidyl-EDDnp	%	K_cat_ (s^-1^)	K_m_ (µM)	k_cat_/K_m_ (s^-1^ µM^-1^)
Cathepsin K		Z-FR-MCA^[7]^	-	-	12.8	9.7	1.31
	-	PAR 1	TLDPRSFLL↓Q	-	2.4	8.5	0.28
	+	**PAR 3**	TLPIK↓TFRGQ	K↓T-96%	2.0	4.3	0.47
	+	PAR 4	LPAP↓**R**↓GYPG↓Q	P↓R-44/R↓G-20/G↓Q-36	1.0	3.3	0.30
Papain		Z-FR-MCA^[33]^	-		42	89	0.47
	-	PAR 1	TLD↓PRSFL↓L↓Q	D↓P-1/L↓L-3/L↓Q-96	0.04	0.5	0.08
	+	**PAR 3**	TLPIK↓TF↓R↓GQ	K↓T-46/F↓R-24/R↓G-30	0.22	1.3	0.17
	+	**PAR 4**	LP↓AP↓**R**↓GYPGQ	P↓A-40/P↓R-48/R↓G-12	0.31	0.9	0.34

Assay conditions: [7] Lecaille et al., 2008; [33] Del Nery et al., 2000. Enrichment of PAR-2 kinetic parameters see Supplementary content, Table S1.

**Table 2:** Kinetic parameters for the hydrolysis of the FRET peptides derived from sequences that span the cleavage sites for activation of protease-activated receptor (PAR) 1,3 and 4 by human cathepsins, L, S, V and B

Enzyme		Peptide	Abz-eptidyl-EDDap	%	k_cat_ (s^-1^)	K_m_ (µM)	K_cat_/K_m_ (s^-1^ µM^-1^)
Cathepsin L		Z-FR-MCA^[27]^	-	-	17.6	1.3	13.5
	-	PAR 1	TL↓DPRSFL↓L↓Q↓	L↓D-30/L↓L-32/L↓Q-16/Q↓Eddnp-22	3.5	2.2	1.6
	+	**PAR 3**	TLPIK↓TFR↓GQ	K↓T-45/R↓G-55	10.6	3.9	2.7
	nh	PAR 4	LPAP**R**GYPGQ	-	-	-	-
Cathepsin S		Z-FR-MCA^[32]^	-	-	2	21	0.09`
	+	PAR 1	TLDPR↓SFLL↓Q	R↓S-24/L↓Q-76	1.2	4.2	0.29
	+	**PAR 3**	TLPIK↓TFR↓GQ	K↓T-36/R↓G-64	0.3	8.1	0.04
	-	PAR 4	LPAP**R**G↓YPGQ	-			
Cathepsin V		Z-FR-MCA ^[27]^	-	-	0.71	6.4	0.11
	-	PAR 1	TLDPR↓SFL↓L↓Q	R↓S-55/L↓L-2/L↓Q-R3	0.01	1.0	0.01
	+	**PAR 3**	TLPIK↓TF↓R↓GQ	K↓T-74/F↓R-15/R↓G-11	0.78	1.9	0.41
	+	PAR 4	LPAP**R**↓GYPG↓Q	R↓G-75/G↓Q-43	-	-	-
Cathepsin B		Abz-FRA (eDnp)K^[34]^	-	-	16.9	13.4	1.33
	nh	PAR 1	TLDPRSFLLQ	-	-	-	-
	nh	PAR 3	TLPIKTFRGQ	-	-	-	-
	nh	PAR 4	LPAPRGYPGQ	-	-	-	-

Assay conditions: [32] Bromme et al., 1994; [27] = Bromme et al., 1999; [34] Almeida et al., 2001. Enrichment of PAR-2 of kinetic parameters see Supplementary content, Table S1

### Distinguished effect of Cat K on thrombin-induced human platelet aggregation

**Fig. 2:**
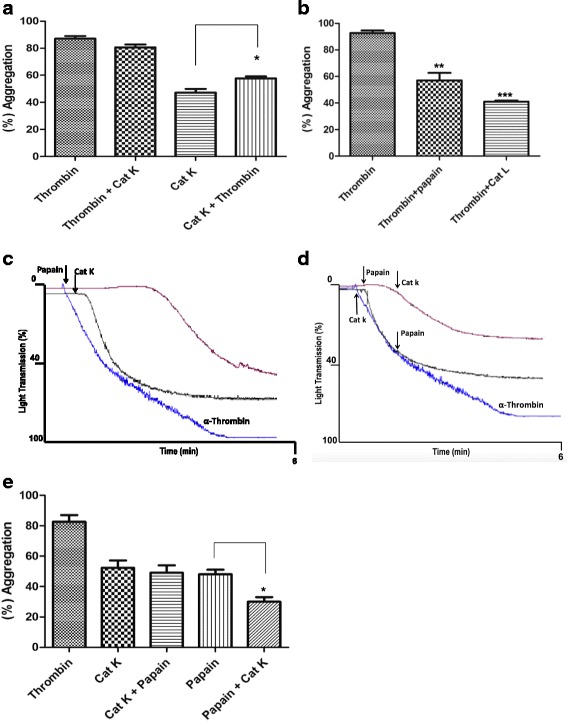
The influence of cat K (20 nM), papain (1.6 µM), and cat L (0.2 µM) on the effect of α-thrombin. (**a**) The results show platelet aggregation induced by cat K (20 nM) followed by α-thrombin stimulation (1.0 UNIH/500 µL) after 2 min. α-Thrombin stimulation was slightly prolonged (black line). Cat K does not interfer with α-thrombin activity on platelets aggregation (blue line). Data are expressed as mean ± SEM from 6 independent experiments (*p<0.05). (**b**) Papain (1.6 µM) followed by α-thrombin stimulation (1.0 UNIH/500 µL) (blue line). The inverse stimulation (α-thrombin followed by papain (1.6 µM) and cat L (0.2 µM)), (black and orange lines). The bar graph shows percentage of aggregation expressed as mean ± SEM from 3 independent experiments (**p<0.01, ***p<0.001). (**c**) Representative traces showing typical aggregation induced by cat K (20 nM), papain (1.6 µM), and α- thrombin (1.0 UNIH/500 µL). The arrows indicate when agonists were added. (**d**) Stimulation by papain (1.6 µM) followed by cat K (20 nM), red line. Inverse cat K stimulation (20 nM) followed by papain (1.6 µM), black line. (**e**) The bar graph shows percentages of aggregation. Data are expressed as mean ± SEM from 3 independent experiments (*p<0.05)

The Cat K pre-stimulation in these washed platelets cells increased α-thrombin promoted platelet aggregation (Fig. 2a, black and green lines). However, Cat K did not affect platelet aggregation when added after α-thrombin (Fig. 2a, blue and red lines).

Conversely, no aggregation was observed when platelets were stimulated by α-thrombin after the addition of cat L (0.2 μM) (Fig. 2b, orange line). The same antagonistic effect was observed with the human cysteine cathepsins V, S, and B (data not shown).

The addition of papain (1.6 μM) after α-thrombin led to a decay in the α-thrombin effect on platelet aggregation (Fig. 2b, blue line). Overall, the maximal aggregation extent in α-thrombin stimulated platelets decreased from 90 % ± 10 to 60 % ± 5, with a *p* < 0.001 (Fig. 2b, black and red lines). The observed decay in α-thrombin-induced platelet aggregation indicates that PAR-4 was papain activated and PAR-1 activity was eliminated, which is the major receptor used by α-thrombin in the process of platelet aggregation.

Because papain and Cat K cleaved PAR-3 and PAR-4 peptides at their activation sites (Lys-Thr and Arg-Gly bonds, respectively; Table 1), the interference of Cat K on the activity of papain in platelet aggregation was assayed. A small decrease in Cat K-induced platelet aggregation was observed when Cat K was added to human platelets before papain (Fig. 2c, d, black and red lines). When Cat K was added 2 min after the addition of papain, a significant decrease of 20 % ± 4 in platelet aggregation was observed (Fig. 2c-e).

### The Cat K effect on platelets is blocked by the PAR-3 antibody and PAR-4 antagonist

The blockage of the Cat K effect was evaluated using PAR-1 and PAR-4 specific antagonists (SCH 79797 and trans-cinnamoyl-YPGKF-NH2, respectively) and PAR-3 antibody, also described as a PAR-3 antagonist [35].

**Fig. 3:**
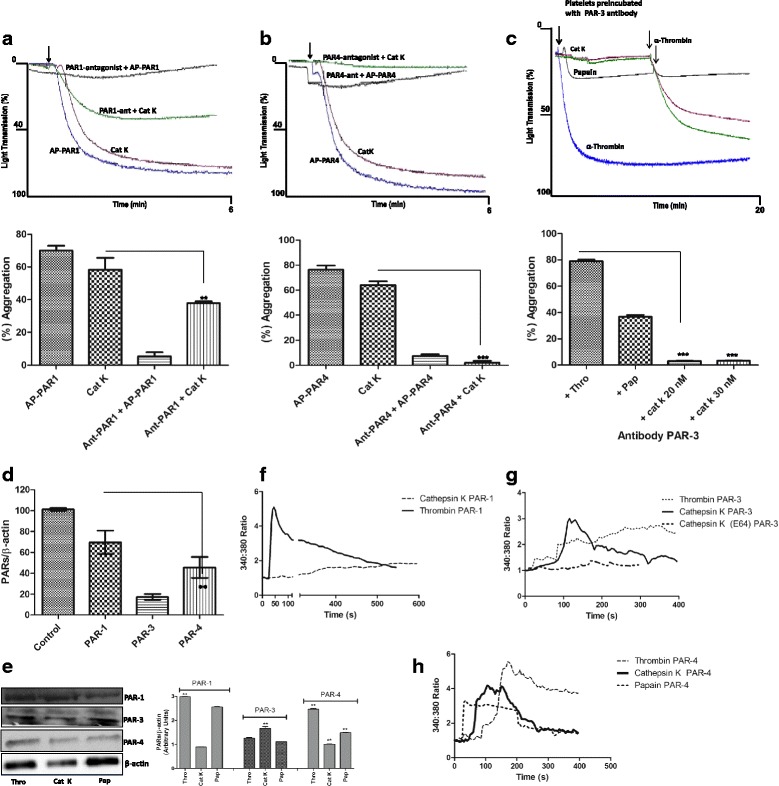
Effect of PAR-1 antagonist (SCH 79797), PAR-4 antagonist (transcinnamoyl- YPGKF-NH2), and PAR-3 antibody on cat K-induced platelet aggregation and intracellular calcium measurements. (**a**) Human platelets were preincubated with the specific PAR-1 SCH 79797 antagonist (140 nM) for 30 min at 37 °C and evaluated in the aggregometer. Representative traces show the interference of SCH 79797 on cat K-induced platelet aggregation. AP-PAR1 (0.2 µM) was completely inhibited. (**b**) The specific PAR-4 trans-cinnamoyl-YPGKF-NH2 antagonist (30 µM) inhibits human platelet aggregation induced by cat K (20 nM). AP-PAR-4 (60 µM) was used as control. (**c**) Inhibitory effect of the PAR-3 antibody (0.02 µM) on cat Kinduced human platelet aggregation. Platelets were preincubated with the PAR-3 antibody for 30 min at 37 °C and aggregation was measured. Representative traces show the inhibition of the cat K effect (red and green lines). Papain-induced human platelet aggregation inhibition, black line. The bar graph shows percentages of aggregation. Data are expressed as mean ± SEM from 3 independent experiments (**p<0.001, *** p<0.0001). (**d**) Reverse transcriptase-PCR using total platelet RNA. Error bars indicate S.D. from triplicate samples. **p <0.01. (**e**). PAR-1, -3, and -4 were detected in washed platelets treated with cat K (20 nM), papain (1.6 µM), and α-thrombin (0.001 UNIH/500 µL) for 10 min at 37 °C; lysate proteins were separated by 10% SDS-PAGE and electrotransferred to nitrocellulose membrane. Membranes were blocked and incubated with anti-PAR1, anti-PAR3, anti-PAR4 rabbit primary antibodies, and anti-ß-actin (control). Antibody binding was visualized by chemiluminescence and the relative levels of these proteins were determined by densitometric analysis. Data are expressed as mean ± SEM from 3 independent experiments (**p<0.001). (**f**) Intracellular calcium measurements in ThrR -/- fibroblasts (absence in PAR-1). Increases in cytosolic calcium in response to cat K (20 nM) and α-thrombin (0.001 UNIH/500 µL). (**g**) ThrR -/- fibroblasts (absence in PAR-3). Increases in cytosolic calcium in response to cat K (20 nM) and α-thrombin (0.001 UNIH/500 µL). Inhibition of human cat K-activity on human platelet aggregation by E-64 (5 µM). (**h**) ThrR -/- fibroblasts (absence in PAR-4). Increases in cytosolic calcium in response to cat K (20 nM), papain (1.6 µM), and α-thrombin (0.001 UNIH/500 µL)

The presence of 140 nM PAR-1 antagonist SCH 79797 decreased the Cat K-induced platelet aggregation by 20 % and, as expected, completely blocked the action of PAR-1-AP (0.2 μM) agonist control (Fig. 3a). The pre-incubation of washed human platelets with the specific PAR-4-antagonist (30 μM) completely abolished the aggregation induced by Cat K (Fig. 3b) and papain (data not shown). In addition, the pre-incubation of human platelets with the PAR-3 antibody (0.02 μM) promoted a complete inhibition of the Cat K-induced platelet aggregation. Moreover, the addition of α-thrombin at the end of the experiment showed that it still promoted platelet aggregation (Fig. 3c). Conversely, the PAR-3 antibody only inhibited approximately 40 % of the papain-induced platelet aggregation. The presence of PAR-3 on the surface of human platelets was confirmed by the detection of PAR-3 transcripts and protein through RT-PCR and Western blot analyses (Fig. 3d, e).

Fibroblast cells from ThrR−/− mice were loaded with fura-2, and ratiometric calcium imaging was obtained after incubation with Cat K, papain, and α-thrombin. The responses and single-cell imaging are shown in Fig. 3f-h. Both Cat K and papain were found to activate PAR-4, however, only Cat K activates PAR-3. The activation of PAR-3 and PAR-4 by Cat K was completely blocked by E-64. Cat K did not activate PAR-1.

### Cat K triggers the activation of Src and promotes p38 Mitogen-activated protein kinase (MAPK) phosphorylation in human platelets

**Fig. 4:**
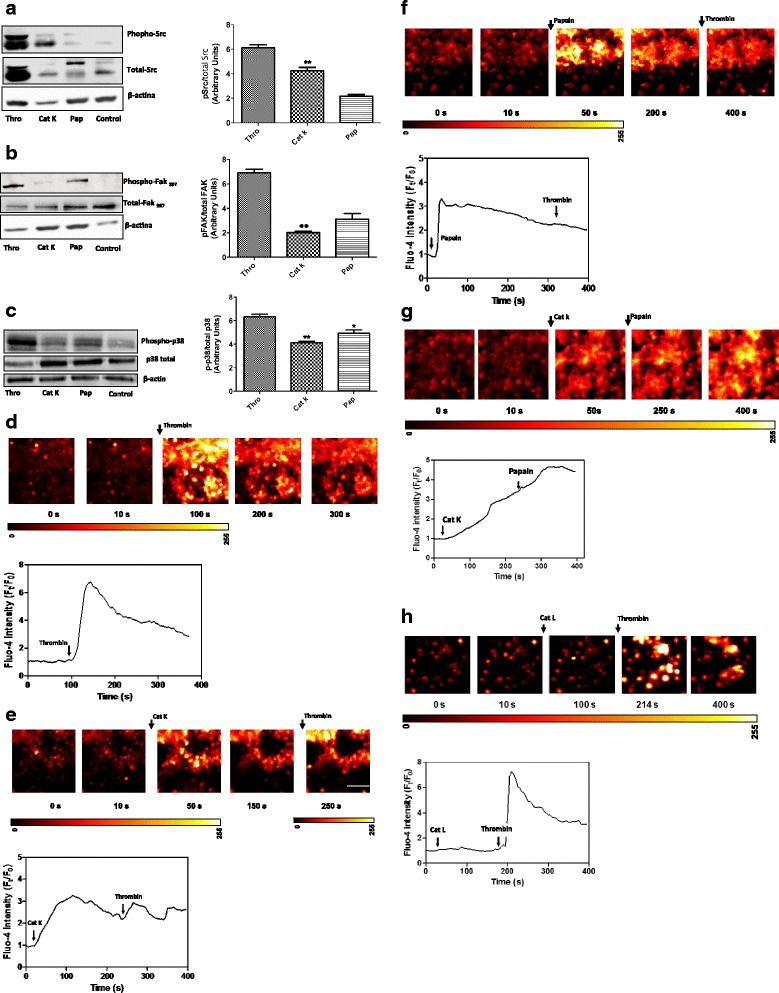
Detection of signaling phosphoproteins by Immunoblot analysis and Ca2+ release into human platelets. (**a**) p-Src family, (**b**) p-FAK, and (**c**) p-p38 washed platelets were treated with cat K (20 nM), papain (1.6 µM), and α-thrombin (0.001 UNIH/500µL) for 10 min at 37 °C; lysate proteins were separated by 10 % SDS-PAGE and electrotransferred to nitrocellulose membranes. Membranes were blocked and incubated with rabbit primary antibodies, anti-phospho-Src (Tyr-416), anti-Src, anti-phospho-FAK (Tyr-397), anti-FAK, anti- phospho-p38 MAPK, anti-p38 MAPK, and anti-ß-actin. Antibody binding was visualized by chemiluminescence and the relative levels of these proteins were determined by densitometric analysis. Papain (1.6 µM) slightly induced Src- and FAK-phosphorylation (2.1 ± 0.2 and 2.5 ± 0.4, respectively). Treatment with α-thrombin resulted in increased Src phosphorylation with the pSrc/total Src ratio of 6.2 ± 0.4, and increased FAK phosphorylation with the pFAK/total FAK ratio of 6.3 ± 0.2. The use of PAR-4 antagonist and PAR-3 antibody eliminated the Src Tyr416 phosphorylation by cat K (unpublished data). The phosphorylation of p38 was required when human platelets were stimulated with cat K. Pre-treatment of platelets with PAR-3 antibody and PAR-4 antagonist abolished the Src-Tyr-416 and FAKTyr- 397 phosphorylation. Data are expressed as mean ± SEM from 3 independent experiments (*p<0.01,**p<0.001, ***p<0.0001). See also Fig. 1a. (**d**) The [Ca2+]i mobilization was measured as described. The arrow indicates when α-thrombin was added to platelets (around 100 s). The washed platelets responded strongly with a transient rise in [Ca2+]i. (**e**) Cat K (20 nM) shows a calcium spike followed by a sustained elevation in the fluorescence ratio. The presence of α-thrombin (1.0 UNIH/500µL) led to an increase in [Ca2+]i indicating that the platelets induced Ca2+ release. (**f**) The papain (1.6 µM) response was similar to that from cat K, however, no substantial rise in [Ca2+]i was detected with the addition of α-thrombin. (**g**) Cat K did not block the papain effect. Sustained elevation in the fluorescence ratio was observed. (**h**) Cat L (0.2 µM) does not induce a detectable effect on Ca2+ mobilization; α-thrombin (1.0 UNIH/500µL) showed a strong response with a transient rise in [Ca2+]i. Platelets were treated with indomethacin (0.1 mg/mL) to prevent thromboxane synthesis, which may also be affected by PKC [Ca2+]. The mesurements were monitored by Fluo-4/AM (4 µM) intensity in a laser scanning confocal miscroscopy; scale bar = 10 µm

The possible involvement of the Src pathway was studied to gain better insights into the signaling involved in the Cat K-induced platelet aggregation. The treatment of washed platelets with Cat K (20 nM) led to Src-Tyr-416 and FAK-Tyr-397 activation. Cat K induced Src-Tyr-416 and slightly induced FAK-Tyr-397 phosphorylation with pSrc/total Src and pFAK/total FAK ratios of 4.04 ± 0.2 and 2.1 ± 0.4, respectively (Fig. 4a, b). As expected, the treatment with α-thrombin resulted in increased Src phosphorylation with a pSrc/total Src ratio of 6.2 ± 0.3, and increased FAK phosphorylation with a pFAK/total FAK ratio of 6.3 ± 0.2 (p ≤ 0.01 and p ≤ 0.001, Fig. 4a, b respectively). Papain (1.6 μM) slightly induced Src- and FAK-phosphorylation (2.1 ± 0.2 and 2.5 ± 0.4, respectively). The use of PAR-4 antagonist and PAR-3 antibody eliminated the phosphorylation of Src Tyr416 by Cat K (data not shown).

We investigated the involvement of p38 MAPK in the process of Cat K activation to gain insight into the biochemical pathway involved in Cat K-induced platelet aggregation. The phosphorylation of p38 was required when human platelets were stimulated with Cat K. Our results demonstrated p38-MAPK phosphorylation by Cat K-stimulated platelet activation (4.2 ± 0.2, Fig. 4c), as observed with α-thrombin (6.32 ± 0.4) and papain (4.62 ± 0.4, Fig. 4c). Under these conditions, a significant p38-MAPK phosphorylation by Cat L, S, and V, which share about 24 to 60 % amino acids sequence identity with Cat K (data not shown), was not observed. This result suggested that the excessive p38 phosphorylation could be a marker for platelet stress through a detectable release of lactate dehydrogenase and enhanced caspase-3 activity (Additional file 1: Figure S1a-c).

### Cathepsin K showed sustained Ca^2+^ mobilization

PARs trigger the transmission of primary activation signals through the phosphorylation of downstream tyrosine or serine/threonine residues in protein kinases. The sequence of kinases phosphorylation is recruited to transmit activating signals of Ca^2+^ release from the contents of platelets, which result in shape alteration, interactions with others platelets and finally clot stabilization. The increase in fluorescence signals generated by stimulating fluo-4-AM loaded washed human platelets with Cat K (20 nM) was used to determine the relative contributions of PAR-3 and −4 activation to the intracellular Ca^2+^ flux; these signals were compared with signals generated by α-thrombin (1.0 UNHI/mL) and papain (1.6 μM). The primary action of α-thrombin through PAR-1 triggered a transient increase in cytoplasmic Ca^2+^ concentration. The average amplitude of F_t_/F_0_ was 6.8 ± 1.0 after the addition of α-thrombin (Fig. 4d). Cat K and papain generated similar heights of calcium increase inducing a prolonged robust response with an average F_t_/F_0_ of 3.3 ± 1.0 for Cat K and 3.4 ± 1.0 for papain (Fig. 4e, f). As indicated in Fig. 4e, the addition of α-thrombin to Cat K activated platelets induced a second increase in cytoplasm Ca^2+^ concentration. However, when α-thrombin was added to the papain preparation, no further Ca^2+^ increase was observed (Fig. 4f).

Thus, Cat K and papain-induced an increase in cytoplasmic Ca^2+^ concentration through a sustained phase and prolonged robust response. However, when platelets were stimulated by Cat K (20 nM), followed by papain (1.6 μM) (Fig. 4g), the effect of papain was maintained. In contrast, Cat L, which shares 60 % similarity with Cat K, did not significantly induce cytoplasmic Ca^2+^ mobilization (Fig. 4h); the subsequent addition of α-thrombin resulted in a robust and transient Ca^2+^ release from dense granules due to the action of α-thrombin through pathways other than PARs activation, such as the GpIb-V-IX complex [18].

### Use of annexin V to detect human platelet activation by Cat K

**Fig. 5:**
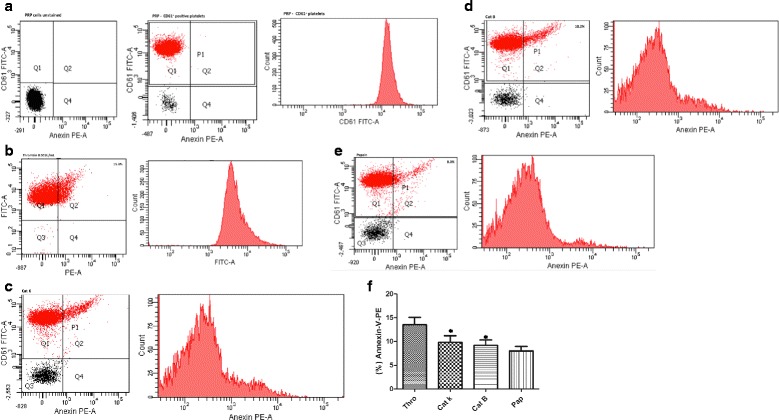
Human platelet activation by cat K. Side and forward scatters of plasma rich in platelets (PRP) from different blood donors prepared under the same conditions. PRP were stained with lineage markers: CD61-FITC (platelets) and Annexin V-PE (phosphatidyserine exposure) or the appropriate isotype controls. PRP was analyzed by flow cytometry. Platelets were treated with apyrase (5.0 UNIH/mL). The areas Q1 and Q2 correspond to nonactivated and activated platelets, respectively. (**a**) Forward - by sidescatter profiles of events in PRP. Populations identified by futher gating. CD61+ nonactivated platelets. Representative histogram of CD61+ platelet resident in PRP. (**b**) α-thrombin (0.001UNIH/mL), (**c**) Platelets activated by cat K (20 nM), (**d**) cat B (0.2 µM), and (**e**) papain (1.6 µM), and their corresponding histograms. Note the increased number of events in Q2 after stimulation with cat K, papain, and α-thrombin. Flow cytometric quantification of Annexin V-PE (beads were used for size calibration). Each figure represents an analysis of 10,000 events with SSC (side scatter) on the abscissa, and FITC fluorescence intensities on the ordinate. (**f**) The bar graph shows percentages of Annexin-V-PE. Data are expressed as mean ± SEM from 3 independent experiments (*p<0.05). See also Fig. 1 and 3

The process of platelet activation is linked to the increased exposure of aminophospholipid phosphatidylserine (PS) on the outer leaflet of the platelet membrane. We tested the effects of Cat K, L, V, S, and B on plasma rich in platelets (PRP) at 37 °C and PS exposure using annexin V-PE. Dot plots of side versus forward angle light scatter for non-activated platelets and platelets labeled with CD61-FITC are shown in Fig. 5a. α-Thrombin (0.001 UNHI/mL) was used as control (Fig. 5b).

Cat K (20 nM) and B (0.2 μM) showed over 10 % ± 5 platelets bound to annexin-V (Fig. 5c-f). Interestingly, papain (1.6 μM) (Fig. 5e, f) activated 8 % ± 3 of platelets and likewise, cat B induced the programmed anuclear cell death process through a detectable release of lactate dehydrogenase (Additional file 1: Figure S1b, c). No significant alteration on PS content exposure was observed after the incubation of platelets with cat V, L, and S (data not shown).

### The induced Cat K platelet dysfunction up-regulates the Hedgehog ligand and growth factors in cells from patients with luminal B breast cancer

To extend these findings to human diseases such as breast cancer, we evaluated the possible correlations between blood coagulation abnormalities, such as platelet activation, and breast cancer cells expressing Cat K during metastasis to bone.

### We investigated Hedgehog ligands in a model based on the co-culture of breast cancer cells and washed human platelets solutions activated by Cat K (20 nM).

**Fig. 6:**
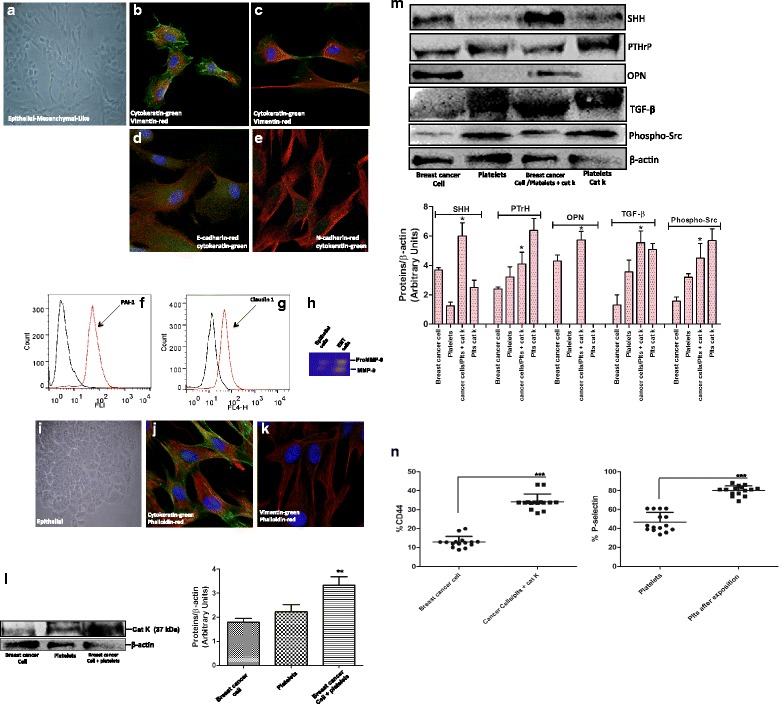
Immunophenotype of breast cancer cells, from patients with Luminal B subtype, used in all experiments. Cells are shown under phase contrast microscopy and indirect immunofluorescence for vimentin, cytokeratin, Ecadherin, N-cadherin, PAI-1, claudin 1, phalloidin, and DAPI (blue, for nuclei). (**a**) Phase contrast–confluent culture of tumor cells with EMT (epithelialmesenchymal- transition) after 3 days. (**b**-**e**) Analysis of epithelial and mesenchymal markers by confocal microscopy; cytokeratin, vimentin, Ecadherin, and N-cadherin, respectively. (**f** and **g**) Representative flow cytometry histograms of PAI-1 and claudin 1 on tumor cells with EMT. The histogram on the left represents a control staining using an isotype-matched control antibody. (**h**) Zymography for MMP-9, in conditioned medium, in EMT and epithelial cells isolated from women with breast cancer. (**i**) Phase contrast— epithelial cells confluent culture after 2 days. (**j** and **k**) Analysis of epithelial marker cytokeratin. We observed the cytoskeletal organization pattern when using phalloidin. (**l**) The expression of cat K is up-regulated in co-cultured breast cancer cells and platelets. Cat K was assessed by immunoblotting. (**m**) Breast cancer cells in epithelial-mesenchimal transition co-cultured with platelets and activated by cat K showed up-regultation of SHH, PTHrP, OPN, and TGF-ß expression, and increased phospho-Src. The graph represents the densitometric analyses from the immunoblotting results. The results are represented as band intensities in arbitrary units relative to the respective total loading control (ß-actin). (**n**) The flow cytometry results are reported as percentages of CD44 human-specific antibody in breast cancer cells, and P-selectin in human platelets activated by cat K after exposure to breast cancer cells. See also Fig. 2

The phenotype of 15 tumor specimens collected from patients with luminal B type breast carcinoma was previously confirmed. Ten of these patients showed different stages in the process of epithelial to mesenchymal transition (EMT) and elevated platelet counts, with an average count of 635 (x 10^3^/μL) and range between 480–844 (× 10^3^/μL) (Additional file 1: Table S2). Epithelial and mesenchymal markers involved in EMT were assayed by immunolocalization, confocal microscopy, and fluorescence-activated cell sorting. Analyses indicated that the cytokeratin (epithelial marker) and vimentin (fibroblast marker) were colocalized on the cell surface (Fig. 6a-c). E-cadherin levels were also reduced in comparison with N-cadherin (Fig. 6d, e). The plasminogen activator inhibitor-1 (PAI-1; Serpine 1, mesenchymal marker) was consistently detected (upregulated); the claudin 1 ephitelial marker was also detected as downregulated (Fig. 6f, g). These results eliminated the hypothesis that breast tumor cells have infiltrating carcinoma-associated fibroblasts, and therefore, confirming the EMT process.

The zymography analysis revealed increased matrix metalloproteinase-9 (MMP-9) secretion (Fig. 6h) suggesting an increased capacity of mesenchymal-like phenotype cells to degrade the extracellular matrix (ECM) and invade the surrounding environment. The remaining five patients with the epithelial phenotype of positive cytokeratin and negative vimentin (Fig. 6i-k) showed normal platelet counts with an average of 290 (x 10^3^/μL) and range between 241 and 395 (× 10^3^/μL) (Additional file 1: Table S2).

Human mesenchymal-like phenotype cells were successfully culture-expanded reaching greater than 90 % confluence in 5 days through weekly passages. The characterization of primary cells was performed on the third passage; this allowed these cells to present a very similar phenotype to that of cells in the original tissues from women with luminal B breast cancer. Mycoplasma contamination was not observed in any of the processed tissues. The expression of Cat K pro-enzyme (37 kDa) was identified in mesenchymal-like phenotype cell extracts from patients with breast cancer and in human platelets; the expression of Cat K was detected through densitometry analysis in both cells in co-culture at 4.2 ± 0.2 (Fig. 6l). The cathepsin cysteine protease activity was detectable in the medium of mesenchymal-like phenotype cells (Additional file 1: Figure S2). Subsequently, mesenchymal-like phenotype cells from women with breast cancer and washed human platelets activated by Cat K were co-cultured. Western blot analysis of these co-cultured cells, after Cat K addition for 24 h, revealed a single band with an apparent molecular mass of 50 kDa, consistent with the presence of SHH in these cells (Fig. 6m). In contrast, SHH was not observed in epithelial cells from patients with breast carcinoma (data not shown). Interestingly, SHH was observed in Cat K-activated human platelets used in co-culture (p ≤ 0.001, Fig. 6m). Unprecedentedly, this cross-talk between the mesenchymal-like phenotype cells and Cat K activated platelets showed SHH expression in human platelets. Furthermore, Cat K activated platelets up-regulated factors that amplify the paracrine Hedgehog signaling. Breast cancer cells in co-culture with Cat K activated platelets played a critical role in up-regulating the expression of PTHrP and Src (p ≤ 0.01, Fig. 6m). The up-regulation in PTHrP expression was 2-fold higher in the mesenchymal-like phenotype cells (5.7 ± 1.2, p ≤ 0.01) than in epithelial cells (3.4 ± 0.4, p ≤ 0.01 – Additional file 1: Figure S4) from breast carcinoma luminal B tissues. Another important protein in the Hedgehog signaling is OPN, which is reported by Das et al. [36] as a “bone metastasized signature”. We observed that unlike Cat K activated platelets, the mesenchymal-like phenotype cells presented increased OPN expression (4.3 ± 0.9, p ≤ 0.01) (Fig. 6m). Conversely, no difference in OPN expression was detected in epithelial carcinoma cells (data not shown). Thus, we investigated whether platelets activated by Cat K had a direct impact on the expression of Src in breast carcinoma cells co-cultured with these platelets for 24 hr. The results obtained using phospho-Src monoclonal antibody showed that platelets activated by Cat K led to a marked increase in Src expression in mesenchymal-like phenotype cells (4.5 ± 1.0, Fig. 6m). The Western blot analysis of the TGFβ expression in Cat K activated platelets showed an evident direct regulation of TGFβ resulting from contact between platelets activated by Cat K and mesenchymal-like phenotype cells (5.56 ± 0.8, Fig. 6m). Finally, we detected elevated levels of CD44 expression in breast carcinoma cells co-cultured with washed platelets activated by Cat K. Consequently, we observed an increased P-selectin expression in human platelets (p ≤ 0.01, Fig. 6n).

## Discussion

Proteases and PARs have crucial roles in hemostasis, inflammation, pain, and cancer. However, the spectrum of proteases that are activated in these conditions is unclear, and their identity, cellular origin, mechanism of action, and causative roles of specifically activated proteases are not defined. Equally, PARs are recognized and activated by proteases such as cysteine cathepsins, which have diverse physiopathological functions. Certain cysteine cathepsins, such as Cat K, L, and B, are secreted as soluble enzymes in pericellular environments where they remain fully active [2, 24, 37, 38].

As previously mentioned, Cat K is a highly potent collagenase with predominant papain-like characteristics that is expressed in osteoclasts. It is present in most epithelial tissues, functions in the lysosomal and extracellular environments as a soluble enzyme, and shows optimal activity at pH between 5.0 and 8.0. This enzyme has been implicated in osteoporosis, arthritis, atherosclerosis, metabolic function, and breast cancer [22, 24, 36, 39, 40].

Platelets, the cells’ chief mediators of hemostasis, are important in these processes, and express three of the four known PARs, PAR-1, −3, and −4. Some studies have demonstrated the action of cathepsins on PARs: the human cat S cleaves PAR-2 in colitis while [38] plant cysteine protease cathepsins (bromelain, ficin, and papain) activate PAR-2 and PAR-4 in itching processes [41, 42].

Thus, we evaluated the ability of the human cysteine cathepsins K, L, B, S, and V to induce human platelet aggregation using papain as a cysteine protease prototype. Our results show that Cat K, but not L, B, S, and V, induced human platelet aggregation in a dose-dependent manner.

Although all five human cathepsins – K, S, L, V, and B – have conserved three-dimensional structure and similar substrate specificity, distinct amino acid preferences that differentiate each enzyme were identified in this study. We used synthetic substrates that mimic the cleavage sites for PARs activation. Cysteine cathepsins - K, S, L, V, and B - generally accept Arg and Lys at the P1 position, which are key amino acids recognized by thrombin when forming tethered ligand sequences for the activation of receptors. Cat L, S, and V clearly show unspecific cleavage sites in the three substrates. In addition, cat L did not hydrolyze the substrate derived from PAR-4. Similarly, cat B did not hydrolyze these substrates but was the only cysteine cathepsin previously investigated in human platelet aggregation. Honn et al. [16] described the association between tumor cells and platelet aggregation using papain as a cat B-mimicking agent and concluded that cat B released from tumor cells induced human platelet aggregation. The crystal structure resolution of cat B [43] showed that human cysteine cathepsins share great sequence similarity, but also contain variable portions that confer important differences in proteolytic specificity and participation in regulatory mechanisms [7, 8].

Unlike the other enzymes tested, Cat K displayed the most distinguishing substrate specificity. Cat K exclusively cleaves PAR-3 and PAR-4 peptides at the Lys-Thr and Arg-Gly bonds, mimicking the activity of α-thrombin on these substrates, and demonstrates its role as possible PAR-3 and PAR-4 agonists. On the other hand, the proteolytic nature of the Cat K effect on human platelet aggregation was demonstrated using the classical cysteine proteinase inhibitors, E-64, LMWK, and HMWK, and correlating the complete inhibition of Cat K activity to the absence of platelet aggregation. This result reinforces the idea that PARs cleaved by Cat K initiate platelet aggregation. Interestingly Cat K did not impair α-thrombin-induced platelet aggregation, indicating that Cat K acts on PARs but does not hinder the PAR-1 receptor. The repeated or continuous exposure of washed platelets to papain and Cat L, V, B, and S alters the PARs response to α-thrombin in such a way that the cellular response is dampened or completely switched off. These results are in agreement with the observed kinetic parameters and show that only Cat K and papain-induced platelet aggregation through PAR-3 and PAR-4 activation, respectively. Similarly, papain does not interfere with the Cat K-induced human platelet aggregation but rather Cat K reduced the papain response. These results indicate that Cat K acts on PAR-3 and PAR-4; however, the effect of papain is only through the activation of PAR-4, as observed by Reddy and Lerner [41].

To reinforce the involvement of PARs-3 and −4 on the effects of Cat K, we used PAR-1 (SCH 79797) and PAR-4 (trans-cinnamoyl-YPGKF-NH_2_) specific antagonists and PAR-3 antibody. Thus, the aggregation of human platelet elicited by Cat K was completely blocked by the PAR-3 antibody and PAR-4 antagonist. Contrary to the expected results, the specific PAR-1 antagonist, SCH 79797, was shown to interfere with Cat K-induced platelet aggregation. Perhaps one of the PARs, such as PAR-3, regulates the PAR-1 signaling through receptor dimerization as described by McLaughlin et al. [44].

The presence of a functional PAR-3 in human platelets remains unclear, however, PAR-3 is also a α-thrombin substrate; its expression in human platelets has been described by others authors [19–21]. PAR-3 is considered a non-signaling receptor and its agonist proteases remain uncharacterized [45]. It is important to mention that our pharmacological assay data were confirmed by the ready detection of PAR-3 mRNA transcripts in human platelets through reverse transcription-PCR. In addition, PAR-1, PAR-3, and PAR-4 were detected by Western blot in washed platelets treated with Cat-K, α-thrombin, and papain; an increased PAR-3 expression was confirmed by densitometry analysis in platelets treated with Cat K. To support these results, we used fibroblast cells from ThrR ^−^/^−^ knockout mice in conjunction with selective antagonists for PAR-1, −3, and −4. No substantial increase in [Ca^2+^]_c_ was detected in ThrR ^−^/^−^ knockout mice compared to that in wild-type mice.

Collectively, these results clearly indicate that Cat K acts as an agonist to PAR-3 and PAR-4 and induces platelet aggregation; moreover, it indicates that the PAR-3 antibody and PAR-4 antagonist inhibited this aggregation.

Hence, we hypothesize that PAR-3 activation by Cat K in human platelets may represent a specific mechanism that drives target cells to express transmembrane signaling triggers in inflammation or cancer processes, such as breast cancer.

Moreover, we do not discard the possibility that PAR-3 is a PAR-4 cofactor and, possibly, a PAR-1 cofactor. The Coughlin research group [45-47] reports that mouse PAR-3 did promote cleavage and activation of human PAR-4 as effectively as it did on mouse PAR-4. Because human platelets express PAR-1 and PAR-4, but much less PAR-3, and because PAR-1 and PAR-4 can independently mediate thrombin signaling, we evaluated the PAR-3 activation and its possible consequent transmembrane signaling.

This investigation was based on the fact that the Src/FAK adhesion focal complex usually includes the first proteins implicated in signaling pathways mediated by PARs −1, −3, and −4 in platelets, e.g. platelets aggregate and adhere to the site of vascular injuries [21, 48]. Our findings confirm an increase in Src-Tyr-416 and FAK-Tyr-397 phosphorylation in washed human platelets stimulated with Cat K. Moreover, focal adhesions act as a signaling center mediating multiple dynamic protein-protein interactions and consequently regulating the assembly and disassembly of focal adhesion sites, such as those with endothelial and others cells, which are essential in the control of platelet movements [49]. It is important to note that the pre-treatment of platelets with PAR-3 antibody and PAR-4 antagonist abolished the phosphorylation of Src-Tyr-416 and FAK-Tyr-397.

It is well established that α-thrombin induces MAPK activation in human platelets. Human platelets contain several members of the MAPK family, including p38. The p38 MAPK activation is observed as a consequence of Src/FAK phosphorylation [49–51]. The role of p38 in platelets is associated with platelet dispersion and cytoskeleton reorganization; the release of Ca^2+^ by dense-granules occurs as a consequence and induces responses for platelet activation and aggregation [49–51]. The increase in p38-MAPK phosphorylation in washed platelets pre-treated with Cat K was confirmed based on the results using α-thrombin as a control. In addition, our data show that, similar to papain, Cat K induces a robust, sustained, and prolonged response to Ca^2+^ increase that is characteristic of PAR-4 activation, and required for the exposure of phosphatidylserine (PS) by platelets and acceleration of platelet activation [42, 52]. Furthermore, the α-thrombin ability to increase transient Ca^2+^ mobilization was blocked in the presence of papain, but not in the presence of Cat K. Interestingly, when we evaluated the effect of papain in the presence of Cat K on platelet aggregation, the sustained phase of Ca^2+^ mobilization was prolonged and similar to that observed in platelet aggregation when Cat K does not block the papain-induced platelet aggregation.

At this point, we investigated platelet functions using washed human platelets. Human platelets were washed to exclude plasma enzymes and prevent undesired platelet functions [53]. Plasma rich in platelets (PRP) was used in the detection of platelet activation because it contains plasma enzymes and interferes with spontaneous aggregation by competing for sites in PARs. We compared different cathepsins and their ability to induce platelet activation through the exposure of the anionic inner PS on the platelet’s surface. Therefore, Cat K and papain-induced human platelet activation were observed by the detection of positive PS-annexin-V. This Cat K ability could be correlated with the enhancement of platelet aggregation induced by the in vitro tumor cells medium; this result indicates a dysregulation between the high activity of cysteine proteases and low levels of their respective endogenous inhibitors, showing their potential metastatic effect in tumor cells in vivo [1, 16, 54, 55].

**Fig. 7:**
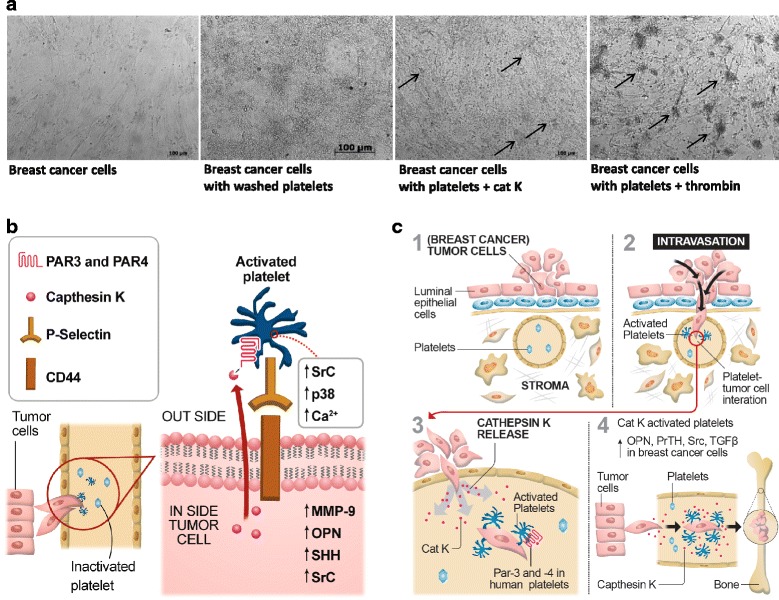
Hypothesized molecular coordination between Cat-K-induced platelet aggregation and crosstalk with epithelial-mesenchimal-like cells from patients with breast cancer. The activation and release of cat K occurs in epithelial-mesenchimal transition cells in breast cancer subtype Luminal B. (**a**) Breast cancer cells cultured with human platelets and activated by cat K and α-thrombin. Phase contrast images of epithelial-mesenchimal-like cells co-cultured with platelets activated by cat K for 24 h. (**b**) Cat K may activate PAR-3 and PAR-4 in human platelets, by receptor cleavage, and trigger platelet aggregation. Activated PAR-3 and PAR-4 can perpetuate the cohesion of tumor cells during heteroaggregation with increase in P-selectin and CD44. The crosstalk between platelets activated by cat K and epithelialmesenchimal tumor cells form microaggreates and promote up-regulation of Hedgehog ligands and growth factors such as SHH, OPN, PTHrP, and TGFß. (**c**) Mammary stroma (1), intravasation (2), cat K secretion by epithelialmesenchimal tumor cells (3), and activate human platelets that could directly affect the expression of the ligands of Hedgehog signaling, reported as an aberrantly activated pathway in breast cancer and related to bone metastasis markers

Our results suggest the involvement of Cat K-induced platelet aggregation in human diseases such as breast cancer. Breast cancer comprises clinically and molecularly distinct tumor subgroups that differ in cell histology and biology and show divergent clinical phenotypes that hamper phase III trials such as those utilizing Cat K inhibitors. We chose the luminal B subtype of breast cancer because it is usually more associated with bone metastasis than other types of breast cancer [54]. Here we correlate epithelial-mesenchymal-like transition cells, from breast cancer patients, with Cat K-induced platelet aggregation (Fig. 7a). Ten of these patients had elevated platelet counts. The co-culture of breast cancer cells with washed human platelets activated by Cat K up-regulates SHH and the expression of factors, such as PTHrP and TGFβ, that amplify the paracrine Hedgehog signaling. In addition, we detected an increase in OPN, which is a component of the “bone metastasis signature” in breast cancer cells, i.e. breast cancer cells that metastasized to the bone show increased OPN expression [36, 40]. We also observed an increase in Src phosphorylation, a central non-receptor tyrosine kinase involved in bone metastasis. Our experiments also show elevated levels of CD44 expression in tumor cells, and P-selectin in washed platelets activated by Cat K in co-culture, corroborating the observed expression of these glycoproteins in metastatic tumor cells. Conversely, CD44 is a marker for stem cell-like cells that indicates cellular heterogeneities and, consequently, is related to worse outcomes, therapeutic resistance, and metastatic progression [55]. Our study reveals that human platelets activated by Cat K-tumor cells with epithelial-mesenchymal-like transition phenotype predominantly up-regulate SHH, PTHrP, TGFβ, Src, OPN, CD44, and P-selectin, and impact the malignancy of breast cancer cells possibly contributing to their tropism to bones.

These results corroborate reports that Cat K is important in platelet-tumor cell cross-talk, which induces platelet aggregation via PAR-3 and PAR-4 (Fig. 7b, c). Moreover, our results enhance the understanding of the molecular coordination between activated platelets and tumor cells, which can support metastasis in secondary sites.

## Conclusion

We concluded that Cat K might activate PAR-3 and PAR-4 mimicking the activity of α-thrombin on these substrates. Cat K-induces platelet aggregation in a dose-dependent manner and triggers Src and p38 phosphorylation and Ca^2+^ influx from the contents of platelets inducing platelet dysfunction, which could facilitate the interaction with breast tumor cells. We have uncovered a mechanism involving a successive series of signaling events linked to Cat K-induced platelet activation and aggregation when co-cultured with breast cancer cells from patients with luminal B carcinoma. With further advances in our understanding of protease functions, origin, temporal release, and properties coupled with improvements in protein engineering, we expect that other therapeutic cysteine protease inhibitors will gain regulatory approval for use in the early stages of breast cancer [56–58]. This therapeutic approach will represent a significant contribution to the treatment of this disease.

## Additional file

Additional file 1:
**Supplemental information includes Supplemental methods, two tables and four figures.** (DOC 1589 kb)

## Abbreviations

PARs: protease activated receptors
ThrR: thrombin receptor
OPN: osteopontin
SHH: sonic hedgehog
PTHrP: parathyroid hormone-related protein
