# Supplementary material for: Cathepsin K induces platelet dysfunction and affects cell signaling in breast cancer - molecularly distinct behavior of cathepsin K in breast cancer
Source: BMC Cancer. 2016 Mar 1;16:173. doi: 10.1186/s12885-016-2203-7 (PMC4774035; doi:10.1186/s12885-016-2203-7)
Supplement: Additional file 1: — Supplemental information includes Supplemental methods, two tables and four figures. (DOC 1589 kb) [file 12885_2016_2203_MOESM1_ESM.doc]

**Additional file 1**

**Cathepsin K Induces Platelet Dysfunction and Affects Cell Signaling in Breast Cancer -Molecularly distinct behavior of cathepsin K in breast cancer**

**Table S1 (related to Tables 1 and 2). Kinetic parameters used in the hydrolysis of FRET peptides derived from sequences that span the cleavage sites involved in the activation of PAR-2 by human cathepsins.**

Assay conditions: [1] = (Lecaille et al., 2008); [2] = (Bromme et al., 1994); [3] = (Bromme et al., 1999); [4] = (Almeida et al., 2001).

**Table S2. Characteristics of Samples Used in the Study**

**Figure S1 (related to Fig. 4 and 5) Pretreatment of platelets with human cat L, V, S, and B – Detection of p38 by Immunoblot Analysis and the LDH Release Test.** (**a**) Washed platelets were treated with cathepsins L, V, S, and B (all enzymes at 0.2 µM), and α-thrombin (1.0 UNHI/mL), and Con A (10 µM) as positive control. Lysates were examined for p38 MAP kinase activation by Western blotting with p38 MAP kinase phosphospecific antibodies. Total p38 MAP-kinase-specific antibody was used to verify equal protein loading. (**b**) Platelet lysis resulted in lactatehydrogenase release and LDH catalysis to convert NADH into NAD^+^. Therefore, increased NADH oxidation, which was measured by change in absorbance at 340nm, was indicative of increased platelets’ lysis and LDH release. (**c**) Analysis of the activation of caspase-3. The caspase activity was investigated by flow cytometry. Flow cytometry analysis of the endogenous levels of activated caspase-3 in washed platelets treated with cathepsins L, V, S, and B (all enzymes at 0.2 µM), and Con A (10 µM, as positive control). The filled and open histograms represent unstimulated and stimulated samples, respectively.


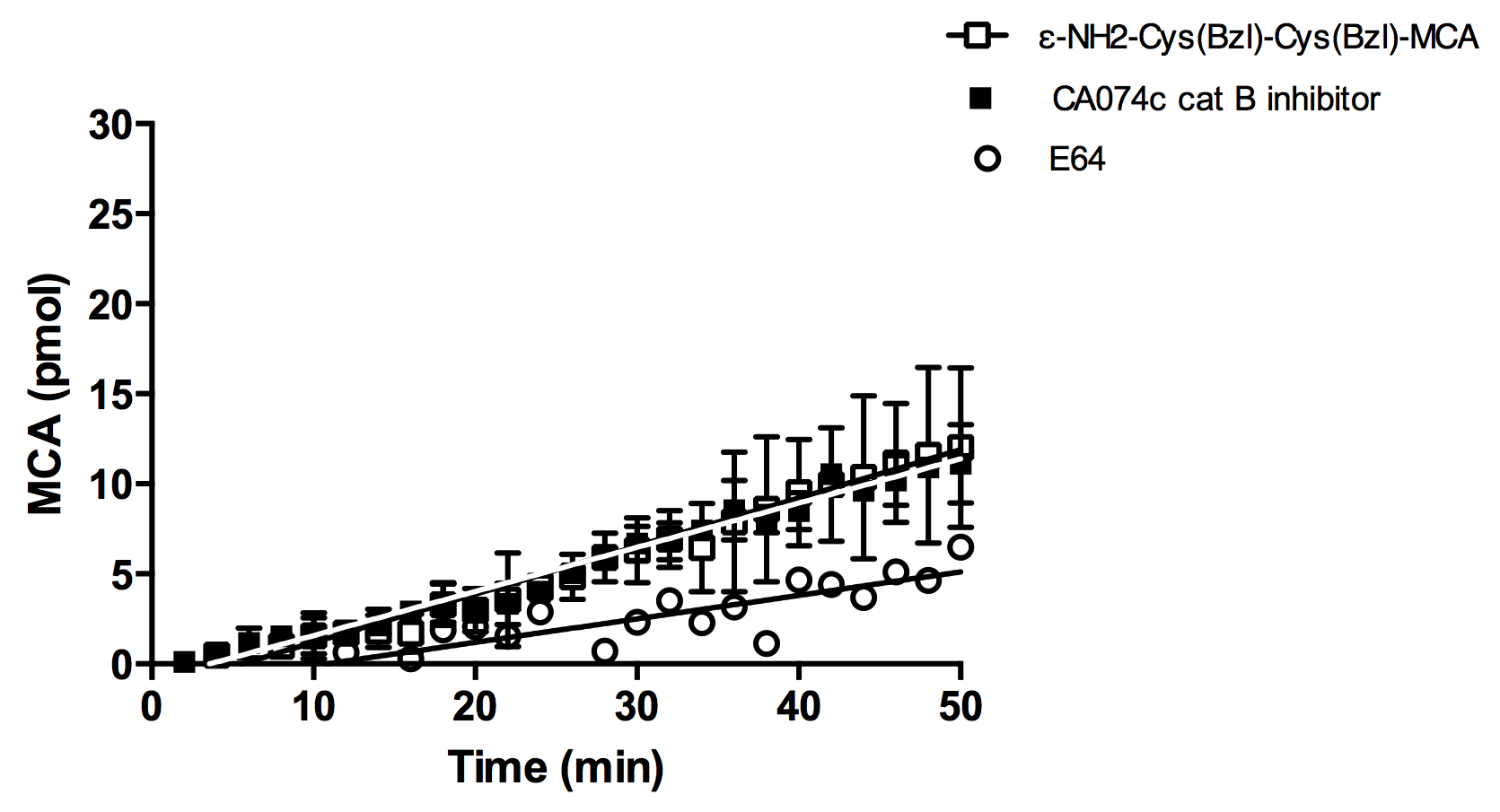


A

B

**
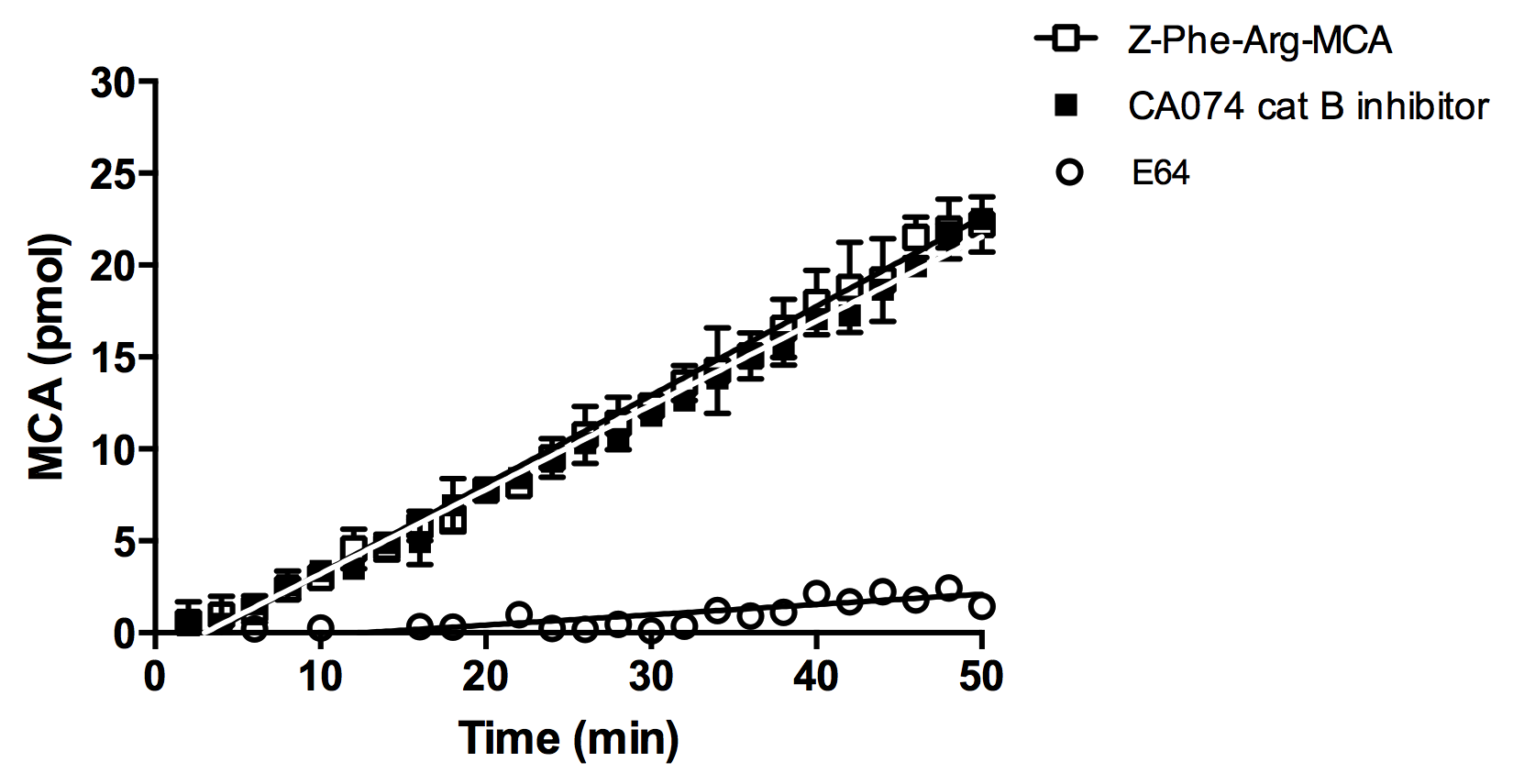
**

**Figure 2 (related to Fig. 6) Cysteine Protease Activities in Media from Epithelial-mesenchymal Cells and epithelial cells (control cells) from Women with Luminal B Subtype Breast Cancer.** Approximately 2 x 10^5^ cells were cultivated for 3 days; enzymatic activities were measured in media (in the absence of phenol red) removed from each well, using (**a**) EMT cells; ε-NH2-caproyl-Cys(Bzl)-Cys(Bzl)-MCA (20 μM), (**b**) EMT cells; Z-FR-MCA (20 μM) (200 μl ﬁnal volume), (**c**) epithelial cells; ε-NH2-caproyl-Cys(Bzl)-Cys(Bzl)-MCA (20 μM) and (**d**) epithelial cells; Z-FR-MCA (20 μM) (200 μl ﬁnal volume). A maximum of 10% substrate consumption was considered, and each point represents the mean ± 95% conﬁdence interval of two replicates. The presence of PMSF did not affect the total activity of proteases. Inhibitor concentrations: PMSF=1 mM; CA074=1 μM; E64=5 μM.

**
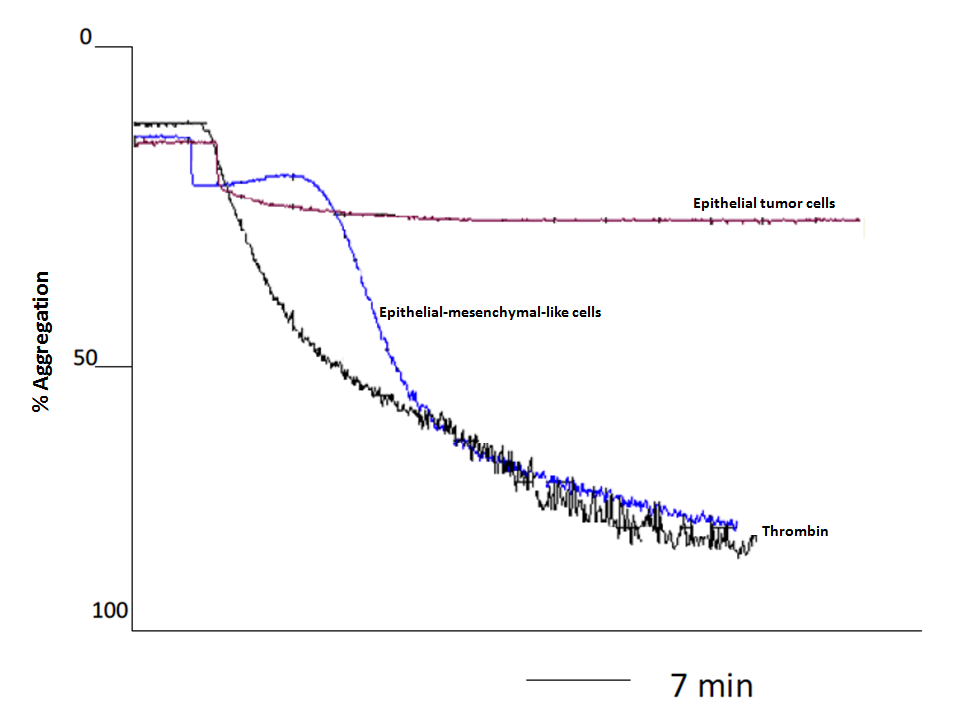
**

**Figure 3 (related to Fig. 5 and 6) Thrombin-induced platelet aggregation, washed platelet method.** Human platelets (250 x 10^3^/µL) were treated with secretome from epithelial and mesenchymal-epithelial-like (100 µg) cells. α-Thrombin (1.0 UNHI/mL) was used as the control.

**Figure 4 Epithelial carcinoma cells co-cultured with platelets and activated by cat K.** Detection of SHH, PTHrP, OPN, and TGF-ß expression, and increased phospho-Src. The graph represents the densitometric analyses from the immunoblotting results.

**Supplementary material and methods**

**Platelet Aggregation**

The effect of human cysteine proteases was studied in 40 healthy volunteers; samples of venous blood (type A, B, and O) from healthy donors were collected into conical plastic tubes containing 3.8% trisodium citrate 1:10 (v/v). Platelet aggregation was measured using washed platelets (250-300 x 10^3^/µL) or platelet-rich plasma (PRP) (200-300 x 10^3^/µL). The procedure used for washing platelets was performed as previously described [5,6]. A 500 µl aliquot of washed platelet suspension was placed in aggregometer cuvettes and incubated at 37 °C for 5 min. Subsequently, agonist solutions containing cysteine cathepsins were separately and individually added to the washed platelet aliquots: cathepsin K (20 nM), cathepsins L, V, S, and B (all enzymes at 0.2 µM), and papain (1.6 µM). The aggregation curve was recorded between 6 and 20 min after the addition of cathepsins and papain. α-Thrombin (1.0 UNIH/500 µl), 0.2 µM activating peptides-PAR1 (AP-PAR-1), or 200 µM AP-PAR-4 were used as agonists for aggregation in washed platelet suspensions. Enzymes preincubated with cysteine inhibitors (E-64 (5 µM), LWMK (1.0 µM), and HWMK (1.0 µM)), and platelets pretreated with PAR-3 antibody and PAR-1 (SCH 79797) and PAR-4 (trans-cinnamoyl-YPGKF-NH_2_) antagonists were tested as blockers for cat K-induced platelet aggregation. Light transmission changes were monitored with an aggregometer (Chrono-Log Corp.) following the method described by Born and Cross [7]. Controls were performed at the beginning and end of experiments.

**Extraction and Real Time Reverse Transcription-PCR Analysis**

Platelets (300 x 10^3^/µL) were separated from white blood cells (WBCs) using abundant amounts of Tyrode solution. Under these conditions, the typical WBC contamination in washed human platelets was approximately 0.01%, which was considered negligible. The sample was then subjected to a hemogram analysis using a semi- automated cell counter KX-21N System (Sysmex America Inc., IL, USA) to ensure enough platelets for RNA extraction and washed platelets free of red blood cells, WBCs, and other cell debris that can occur following a low-speed centrifugation step. RNA extraction was performed according to the TRIzol®LS (Life Technologies, Carlsbad, CA, USA) protocol with minor modifications to enhance total RNA yield. The quality and quantity of the extracted total RNA were superficially assessed on 1% agarose gel. RNA was resuspended in 50% deionized formamide and run on a 1% agarose gel buffered with 0.5 Tris–Borate–EDTA (TBE). The concentration and purity of RNA samples were assessed by the 260 and 280 nm readings and ratios. First strand cDNA was reverse transcribed using 1.0 μg of total RNA and the ImProm-II^TM^ reverse transcription kit (Promega, Madison, WI, USA), according to the manufacturer’s protocol. Quantitative RT-PCR amplification was performed on 2 µl of cDNA (1:5) using specific primers for PAR-1 forward, 5’–TGCTTCAGTCTGTGCGG-3’ and; reverse, 5’-GGGATCTAAGGTGGCATTTGT-3’; PAR-3, forward, 5’-AGTTCCCCTTTTCTGCCTTG-3’ and; reverse, 5’-CATGGAGATGTGAAGCACTTTC-3’, and PAR-4 forward, 5’-ACAGCACGCCCTCAATC-3’ and; reverse, 5’-AGGGTGTCACTGTCATTGG-3’ and the SYBR Green Master Mix kit (Applied Biosystems, Foster City, CA) in a 7500 Real-Time PCR System (Applied Biosystems, Warrington, UK). The cycling conditions were 95 °C for 10 min and 30 cycles of 95 °C for 15 s, 60 °C for 1 min, and 72 °C for 30 s. The specificity of the amplified products was analyzed through dissociation curves generated by the equipment yielding single peaks. Negative controls were used in parallel to confirm the absence of contamination. Data were analyzed using the 2^-∆Ct^ method and the 7500 Real-Time PCR System software. The expression values were normalized to β-actin [8].

**Protein preparation and Immunoblot analysis**

Washed platelets (200 x 10^3^/µL) were treated with cathepsins, cysteine proteinases K (20 nM), L, V, S, and B (all enzymes at 0.2 µM), and papain (1.6 µM) and α-thrombin (0.001 UNHI/mL) for 10 min at 37 °C, for the detection of PARs -1, -3, and -4 and extraction of signaling phosphoproteins (p-PKC, p-SOD, p-Src family, p-FAK, and p-p38). Pellets of washed platelets were obtained by centrifugation at 880 x g for 15 min, lysed in ice cold lysing buffer (20 mM Tris, 300 mM NaCl, 2 mM EGTA, and 2% NP-40 (nonidet-P40)) at pH 7.5 containing the Roche Complete Protease Inhibitor Cocktail (Basel, Switzerland), phosphatase inhibitors, 1 mM Na_3_VO_4_ (sodium orthovanadate), and 100 mM NaF (sodium fluoride), and frozen at -80 °C. Total protein content was measured using the Micro BCA Protein Assay kit from Pierce (Rockford, IL, USA). Proteins were transferred to GE Healthcare nitrocellulose membranes (Pittsburg, PA, USA) by 2.4-h electroblotting at 200-mA constant current in blotting buffer (20 mM Tris base, 150 mM glycine, and 20% methanol), using the Mini Trans-Blot Electrophoretic Transfer Cell from Bio-Rad (Hercules, CA, USA). The membranes were quenched for 2 h with 1% to 0.1% bovine serum albumin (BSA) in TBST buffer (200 mM Tris/HCl buffer pH 8.0 containing 150 mM NaCl and 0.05% Tween 20), and incubated overnight at 4 **°**C with anti-PAR1 primary rabbit antibodies from Invitrogen (Carlsbad, CA, USA), anti-PAR3 from Santa Cruz Biotechnology (Santa Cruz, CA, USA), anti-PAR4 from Invitrogen (Carlsbad, CA, USA), anti-Src rabbit antibody (Tyr-416), anti-phospho-Src rabbit antibody, anti-FAK rabbit antibody (Tyr-397), anti-phospho-FAK rabbit antibody, anti-p38 MAPK rabbit antibody, anti-SHH rabbit antibody, anti-PTHrP rabbit antibody, anti-Cat K rabbit antibody, anti-OPN rabbit antibody, anti-TGFβ mouse, and anti-β-actin; all from Cell Signaling Technology (Beverly, MA, USA) and diluted in 1% BSA in TBST. After this incubation, the membranes were sequentially washed three times with TBST. The detection of the chemiluminescence signal was performed using the ECL gel documentation system (UVITEC, Cambridge, UK) and Super Signal from Pierce (Rockford, IL, USA). The densitometry analysis was performed in the Image J software (NIH, USA) using β-actin as the control for each sample.

**Calcium Mobilization by Confocal Microscopy**

Washed human platelets (200 x 10^3^/µL) were incubated with 4 µM Fluo-4/AM (Molecular probes, Invitrogen, USA) at room temperature for 30 min. After incubation, washed platelets were centrifuged at 141 x g for 5 min for subsidence to coverslips (12 mm diameter) and then maintained in Tyrode solution. The cytoplasmic Ca^2+^ concentrations ([Ca^2+^]_i_) were measured after centrifugation. Briefly, platelets were stimulated with cathepsins K (20 nM), L, V, S, and B (all enzymes at 0.2 µM), and papain (1.6 µM) and α-thrombin (1.0 UNIH/mL); the Fluo-4/AM was excited with an argon laser (λ_Ex_ = 488 nm) and the light emission was detected using a Zeiss META detector (λ_Em_ = 500−550 nm). The pinhole device was not used. Images were collected at approximately 5 s intervals. All images were captured and processed using a LSM 510 META confocal microscope (Zeiss, LSM 780, Germany) with a 63 × objective (Plan-Neofluar, 1.4 numerical aperture) under oil immersion. Fluorescence intensity was normalized to the basal fluorescence using the Examiner 3.2 (Zeiss, Germany) and Image J (NIH, USA) softwares.

**Lactate dehydrogenase assay and determination of caspase-3 activation by flow cytometry**

The viability of washed platelets was measured in the presence of cat K (20 nM), cathepsins L, V, S, and B (all enzymes at 0.2 µM), and papain (1.6 µM), Con A (10 µM), and α-thrombin (1.0 UNIH/mL). The LDH activity in lysed platelets (200 x 10^3^/µL) was measured with Triton X-100. After 10 min of incubation and Tyrode washing, a surface sample was immersed in 40 µL of Triton X-100 (diluted to 1%). After 5 min, 25 µL lysates were taken from the surface and mixed in a 96-well plate with 200 µL of substrate solution containing nicotinamide adenine dinucleotide (0.28 mM, NADH); sodium pyruvate (0.32 mM in phosphate buffer, pH 7.4) was used as the substrate. LDH catalyzes the conversion of NADH to NAD^+^. The lysate LDH activity was determined by the change in absorbance at 340 nm detected using a microplate reader. This activity was normalized using a pool of all platelet lysates in the initial washed platelets. The released LDH was expressed as a percentage of the total LDH activity (extracellular + intracellular LDH). Simultaneously, caspase-3 activation was measured using flow cytometry. At the end of the treatment with cathepsins, washed platelets were transferred to cytometry tubes and fixed with 100 μl of 2% paraformaldehyde (v/v) for 30 min at room temperature. The platelets were resuspended in 200 μl of 0.01% glycine in PBS and incubated for 15 min at room temperature. The platelets were then resuspended in 200 μl of 0.01% saponin in PBS and incubated for 15 min at room temperature. Finally, the platelets were incubated with 10 μl of anti-cleaved caspase-3 conjugated with Alexa Fluor 488 (BD Biosciences) for 40 min. The results were analyzed by flow cytometry (Accuri C6; BD Biosciences).

**Enzyme activities**

The activities of cysteine proteases were quantified through fluorometric assays using either carbobenzoxy-Phe-Arg-7-amide-4-methylcoumarin (Z-FR-MCA, Sigma-Aldrich Corp., St. Louis, MO, USA) or ε-NH_2_-caproyl-Cys(Bzl)-Cys(Bzl)-MCA (synthesized by Prof. Dr. Maria A. Juliano) [9] as substrates. These substrates were used to quantify total cysteine-proteases and cat B, respectively. The incubations were carried out in black microplates (Corning, MA, USA), in 50 mM phosphate buffer at pH 6.3 containing 10 mM EDTA. The enzymes (100 μg of protein) were pre-activated by incubating media aliquots with 2 mM dithiothreitol (DTT, 10 min, room temperature) and the subsequent addition of substrate (20 μM, 200 μl final volume). The fluorescence produced upon the hydrolysis of substrates was measured every 20 seconds in FlexStation 3 (Molecular Devices, CA, USA) using λ excitation = 380 nm and λ emission = 460 nm. The assays were also performed in the presence of the following inhibitors: 1 mM phenylmethylsulfonyl fluoride (PMSF, inhibitor of serine-proteases), 5 μM E64 (irreversible inhibitor of cysteine-proteases), and 1 μM CA074 (irreversible inhibitor of cat B).

**Supplementary references**

1. Lecaille F, Bromme D, Lalmanach G. Biochemical properties and regulation of cathepsin K activity. Biochimie. 2008;90:208-226. doi:10.1016/j.biochi.2007.08.011.

2. Bromme D, Smith RA, Coles PJ, Kirschke H, Storer AC, Krantz A. Potent inactivation of cathepsins S and L by peptidyl (acyloxy)methyl ketones. Biol Chem Hoppe Seyler. 1994;375:343-347. DOI: 10.1515/bchm3.1994.375.5.343.

3. Bromme D, Li Z, Barnes M, Mehler E. Human cathepsin V functional expression, tissue distribution, electrostatic surface potential, enzymatic characterization, and chromosomal localization. Biochemistry (Mosc). 1999; 38:2377-2385. DOI: 10.1021/bi982175f.

4. Almeida PC, Nantes IL, Chagas JR, Rizzi CC, Faljoni-Alario E, Carmona A, Juliano L, Nader HB, Tersariol IL. Cathepsin B activity regulation. Heparin-like glycosaminoglycans protect human cathepsin B from alkaline pH-induced inactivation. J Biol Chem. 2001;276:944-951. 10.1074/jbc.M003820200.

5. Isenberg JS, Romeo MJ, Yu C, Yu CK, Nghiem K, Monsale J, Rick ME, Wink DA, Frazier WA, Roberts DD. **T**hrombospondin-1 stimulates platelet aggregation by blocking the antithrombotic activity of nitric oxide/cGMP signaling. Blood. 2008;111: 613-623. doi: http://dx.doi.org/10.1182/blood-2007-06-098392.

6. Andrade SS, Silva MC, Gouvea IE, Kondo MY, Juliano MA, Sampaio MU, Oliva ML. Baupain, a plant cysteine proteinase that hinders thrombin-induced human platelet aggregation. Protein Pept Lett. 2012;19:474-477. doi: 10.2174/092986612799789305.

7. Born GV, Cross MJ. The Aggregation of Blood Platelets. J Physiol. 1963;168:178-195. DOI: 10.1113/jphysiol.1963.sp007185.

8. Livak KJ, Schmittgen TD. Analysis of relative gene expression data using real-time quantitative PCR and the 2(-Delta Delta C(T)) Method. Methods. 2001;25:402-408. doi:10.1006/meth.2001.1262.

9. Alves LC, Almeida PC, Franzoni L, Juliano L, Juliano MA. Synthesis of N alpha-protected aminoacyl 7-amino-4-methyl-coumarin amide by phosphorous oxychloride and preparation of specific fluorogenic substrates for papain. Pept Res. 1996; 9:92-96.
